# Performance of ICP-TOF-MS for ultra-trace element analyses in ice cores

**DOI:** 10.1039/d5ja00286a

**Published:** 2025-10-06

**Authors:** Tatjana S. Münster, Theo M. Jenk, Anja Eichler, Geunwoo Lee, Margit Schwikowski

**Affiliations:** a PSI Center for Energy and Environmental Sciences Villigen PSI Switzerland theo.jenk@psi.ch; b Department of Chemistry, Biochemistry and Pharmaceutical Sciences, University of Bern Bern Switzerland; c Oeschger Centre for Climate Change Research, University of Bern Bern Switzerland; d Climate and Environmental Physics, Physics Institute, University of Bern Bern Switzerland

## Abstract

Ice cores serve as unique paleo-archives allowing access to long-term records of trace elements, which are important for our understanding of global biogeochemical cycles and air pollution. However, analysis of trace elements in ice cores is a challenge, because of very low concentration levels and their presence in dissolved and particulate form. The commonly used and well-established technique for analysis of trace elements in ice cores is inductively coupled plasma sector field mass spectrometry (ICP-SF-MS). Recently, inductively coupled plasma time of flight mass spectrometry (ICP-TOF-MS) was introduced as a new, promising technique. Due to its fast acquisition time for the near-full mass spectral range, it allows for the detection of short transient signals, offering the opportunity to determine the elemental composition of single particles. In this study, the performance of this new technique for analyzing total trace element concentrations was tested and compared to the one established in the field of ice core research. The focus was on sensitivity, precision, and optimization of sample preparation in sections from two ice cores (Colle Gnifetti, Cerro Negro), characterized by different impurity levels. The performance of the ICP-TOF-MS was excellent for the investigated trace elements within the nominal mass range from 23 to 238, except for ^45^Sc because of insufficiently resolved mass interferences. This is very promising for many applications in ice core research, especially in view of the great benefit to analyze single particles simultaneously in the same sample.

## Abstract 2

Ice cores record trace elements of the past, key for studying biogeochemical cycles and pollution histories. For the challenging ultra-trace analysis, no significant difference in precision or accuracy were detected between ICP-TOF-MS and ICP-SF-MS.

## Graphical abstract

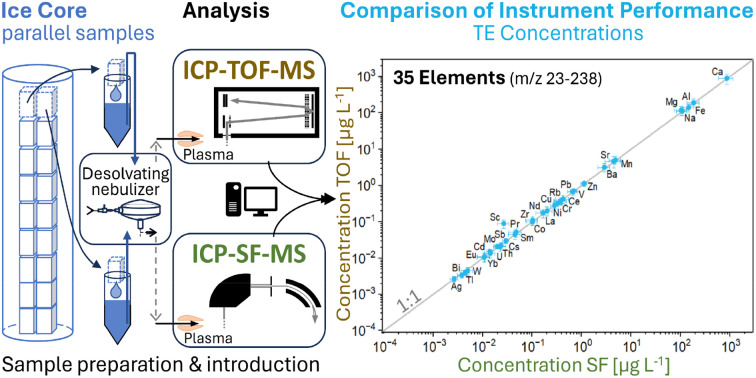

## Introduction

Ice cores serve as unique paleo-archives allowing access to long-term records of trace elements (TEs), which are important for our understanding of global biogeochemical cycles^1^ and air pollution.^2,3^ The underlying assumption is that individual TEs are characteristic for specific sources of the deposited aerosol particles. Sources can be the oceans (sea salt containing *e.g.* Na, Mg),^4^ volcanoes (*e.g.* S, Bi, Tl),^5^ anthropogenic pollution (*e.g.* heavy metals like Pb or Cd)^3,6^ or desert areas (mineral dust containing crustal/rare earth elements).^7,8^ The commonly used and well-established technique for the analysis of TEs in ice cores is inductively coupled plasma sector field mass spectrometry (ICP-SF-MS).^9–11^ The advantage of high sensitivity, mass resolving power and low background is ideal for ice core samples characterized by very low impurity concentration levels in the ng L^−1^ range. However, TE analysis in ice cores is a challenge, not only because of the very low concentration levels, but also because they are present in dissolved and particulate form. While general trends and relative variations over time are typically reproducible, concentration levels of TEs from different studies can often not be compared directly, since they strongly depend on the treatment of the samples.^12–14^ Acidification used to prevent adsorption of TEs on the walls of sample vials, causes a partial leaching of TEs. The amount of leached TEs is influenced by their position within the crystal lattice and the mineral structure.^11^ Feldspars for example, characterized by a complex silicate structure, exhibit strong bonding of TEs, leading to slow dissolution. In contrast, sheet silicates such as muscovite possess weaker interlayer bonds, enabling faster dissolution of TEs into the solution.^14^ Thus, longer acidification times, higher acid strengths and higher particle amounts will lead to increased concentrations.^11,12,14^ To reach an equilibrium between the dissolved and particulate fraction (*i.e.*, near complete digestion), some studies used acidification times of days to weeks.^11,12,14^ All these findings point to the strong dependence of TE partitioning between the liquid and particle phase on the applied conditions, potentially combined with losses of particles in the inlet system including nebulization, and/or limited efficiency in particle evaporation in the plasma.

Inductively coupled plasma time of flight mass spectrometry (ICP-TOF-MS) was introduced for TE analyses in the 1990ies,^15,16^ and was recently made commercially available again.^17^ The TOF mass spectrometer is capable of recording full mass spectra of short transient signals due to its fast acquisition rate (*i.e.* short dwell time) in the range of milliseconds,^18^ compared to seconds typical for ICP-SF-MS scans. This opens up the possibility to investigate the elemental composition of single particles.^18–20^ The potential of ICP-TOF-MS for single particle analysis has already been demonstrated, *e.g.* for engineered nanoparticles in consumer products or nanoparticles in chemical mechanical planarization suspensions,^21–23^ and in a more recent study was also investigated for engineered particles in the micrometer range.^24^ Such nanoparticles usually consist of a single element or a single element plus oxygen, whereas particles in ice cores have a more complex composition. A first application of ICP-TOF-MS for TE analyses in ice cores showed an overall good agreement for the concentrations of Na and Ca with the previously used methods, *i.e.* absorption and fluorescence spectroscopy, respectively.^19^ Except for the latter two elements, which are present predominantly in the dissolved form, the performance of the ICP-TOF-MS for TE analyses in ice core samples has not been characterized in detail.

The motivation of this study is to compare total TE concentrations in glacier ice samples determined by ICP-TOF-MS and the established ICP-SF-MS technique. The goal was to assess whether the newer ICP-TOF-MS technique, with its added capability for single-particle analysis, can also achieve accuracy and precision in ultra-trace element concentration measurements comparable to those traditionally obtained with ICP-SF-MS. We thereby focus on limits of detection (LoD), precision, and the comparability of absolute values and trends in TE concentrations. The ice samples were prepared from ice cores extracted from two glaciers, Cerro Negro (CN; Andes, Chile) and Colle Gnifetti (CG; Alps, Switzerland). The Cerro Negro glacier is heavily affected by melt processes and TE concentrations are generally low. The Colle Gnifetti is a cold glacier, and samples were taken from a section containing both (visibly) clean ice and mineral dust layers originating from the Sahara. The samples from these two glaciers therefore cover a wide range of concentration levels and a high variety in the TE composition. Accounting for the fact that the two ICP instruments are located at different institutions, we first optimized ice sample preparation with respect to acidification time and the possibility of sample preservation prior to the therefore required transport. This allowed to ensure comparable sample conditions, avoiding any potential bias unrelated to the instruments. Since the ultimate goal of future studies using the ICP-TOF-MS is to also determine the TE composition of the single particles in the ice samples, we here applied very short acidification times for minimal dissolution. Accordingly, the acidification related effects on concentration levels were investigated on much shorter timescales than in previous studies.^11,12,14^

## Material and methods

### Samples and sample preparation

The Cerro Negro glacier, located in the Chilean Andes, is a temperate glacier and strongly influenced by melting in summer.^25^ Samples were collected from core sections covering the depth from 1.54 to 7.43 m. The Colle Gnifetti glacier in the Swiss Alps is a cold glacier and samples were taken from 16.78–18.15 m depth of a core collected in 2021.^26^

For both cores, parallel samples from each depth were prepared identically for analysis with the two ICP mass spectrometers as follows. Ice samples were cut in the −20 °C cold room of the PSI, using a stainless-steel band saw with a Teflon coated saw guide and table surface. To remove potential contamination from drilling and core handling, an inner stick with a cross section of 1.6 × 1.6 cm was cut in segments with a sampling resolution of 4.4 to 6.0 cm for the Cerro Negro core (*n* = 112) and of 5.5 cm for the Colle Gnifetti core (*n* = 26). For the Colle Gnifetti core, the two parallel samples were obtained at the same time. This was different for the Cerro Negro core, where the parallel samples were cut in two different campaigns more than two years apart, resulting in reduced precision regarding the exact depth alignment. After cutting, samples were additionally scraped with a ceramic knife to remove around 1 mm of the cutting surface and the saw dust, both potentially contaminated by the sawblade.^9,27^ The decontaminated samples were then stored in 50 ml polypropylene tubes (Sarstedt, Nümbrecht, Germany), pre-cleaned by rinsing five times with ultrapure water (UPW; 18 MΩ cm quality, Mili-Q^®^ Element system, Merck Millipore, Burlingon MA, USA).

Parallel to the samples, artificial ice blanks prepared from UPW were cut. All samples and blanks were taken out from the freezer 1.5 h before analysis and rinsed with UPW for final decontamination. Afterwards they were transferred into 50 ml polypropylene tubes (pre-cleaned by soaking for three days in 0.2 M ultrapure HNO_3_; Optima™, Fisher Chemical, Loughborough, UK), then placed in a water bath at around 25 °C for melting and 1.5 min before analysis were acidified with ultrapure HNO_3_ to a final concentration of 0.2 M. Within that time, they were transferred into 1.5 ml polystyrene auto-sampler vials (Sarstedt, Nümbrecht, Germany, precleaned three times with 0.2 M ultrapure HNO_3_), which were spiked before with 3 μl ^103^Rh-standard (TraceCERT, Sigma-Aldrich, Germany), leading to a final concentration of 0.01 μg L^−1^ for analysis. For the ICP-TOF-MS, the ^103^Rh-standard was continuously introduced into the sample stream, obtaining a final concentration of 0.5 μg L^−1^.

For examining the response of TE concentrations to different acidification times ranging from 1.5 min to 4 h (using ultrapure HNO_3_), aliquots of an ice sample from the Cerro Negro glacier core were analysed with ICP-SF-MS. Additionally, six samples from the Cerro Negro core were re-frozen after acidification and re-analysed one week later.

### Analytical instruments

The two instruments used were an ICP-SF-MS (Element 2, Thermo Fisher Scientific, Bremen, Germany)^28^ located at the Paul Scherrer Institute (Villigen, Switzerland) and an ICP-TOF-MS (icpTOF R, TOFWERK AG, Thun, Switzerland) at the University of Bern (Climate and Environmental Physics; Switzerland), about 100 km apart.

The excellent mass resolution of the ICP-SF-MS of up to *m*/Δ*m* = 10′000 allows for high sensitivity and a low noise level.^10,28,29^ In our application, after a take-up time of 1.5 min to reach signal stabilization, the sample was measured for 3.8 min.

First, 14 scans (*i.e.*, measurements per mass peak) over the complete mass spectrum, from mass-to-charge ratio (*m*/*z*) 7 to 254, were acquired in low resolution (*m*/Δ*m* = 300), followed by 14 scans of a narrower mass range (*m*/*z* 7–103) in medium resolution (*m*/Δ*m* = 4′000). The acquisition time per scan for the complete mass spectrum in low resolution was 9 s (7 s for the *m*/*z* range in mid resolution), with the automatic switch between resolutions requiring 5 s. Note that for subsequent data processing and evaluation, only the raw data from the higher-resolution scans (medium resolution) were used for the *m*/*z* range of 23 to 66. The instrument was tuned daily to optimize sensitivity, signal stability and peak separation by adjusting gas flow rates and ion-optics settings. Also, each day, a mass calibration was performed. Due to known limitations of the ICP-TOF-MS for low masses (see below), only 35 TEs within the *m*/*z* range of 23–238 were analysed (Table 1).

**Table 1:** Mass resolution applied or achieved for 35 TEs analysed by ICP-SF-MS and ICP-TOF-MS, respectively

ICP-SF-MS	Low resolution (*m*/Δ*m* = 300)	^85^Rb, ^88^Sr, ^90^Zr, ^95^Mo, ^103^Rh^a^, ^109^Ag, ^111^Cd, ^121^Sb, ^133^Cs, ^138^Ba, ^139^La, ^140^Ce, ^141^Pr, ^146^Nd, ^147^Sm, ^153^Eu, ^172^Yb, ^182^W, ^205^Tl, ^208^Pb, ^209^Bi, ^232^Th, ^238^U
	Medium resolution (*m*/Δ*m* = 4′000)	^23^Na, ^24^Mg, ^27^Al, ^44^Ca, ^45^Sc, ^51^V, ^52^Cr, ^55^Mn, ^56^Fe, ^59^Co, ^60^Ni, ^63^Cu, ^66^Zn, ^103^Rh^a^
		
ICP-TOF-MS	*m*/Δ*m* = 1′800–2′000	^121^Sb, ^133^Cs, ^138^Ba, ^139^La, ^140^Ce, ^141^Pr, ^146^Nd, ^147^Sm, ^153^Eu, ^172^Yb
	
	*m*/Δ*m* = 2′000–2′500	^23^Na, ^24^Mg, ^27^Al, ^44^Ca, ^51^V, ^52^Cr, ^55^Mn, ^56^Fe, ^59^Co, ^103^Rh^a^, ^88^Sr, ^109^Ag, ^111^Cd, ^182^W
	
	*m*/Δ*m* = 2′500–3′000	^45^Sc, ^60^Ni, ^63^Cu, ^85^Rb, ^90^Zr, ^95^Mo, ^205^Tl, ^208^Pb, ^209^Bi, ^232^Th, ^238^U
		
	*m*/Δ*m* > 3′000	^66^Zn

a Internal standard.

The ICP-TOF-MS was equipped with a collision cell (Q-Cell, filled with 7% H_2_ in He gas). The cell reduces argon interferences and leads to an improved mass resolution.^19^ After daily performed instrument tuning, ICP-TOF-MS analyse was conducted with achieved mass resolutions of *m*/Δ*m* = 1′800–3′100 (Table 1). No specific tuning to optimize for low *m*/*z* values was applied, as this would come at the cost of lower sensitivity for TEs with *m*/*z* above ∼150.^30^ Therefore, the mass range measured by ICP-TOF-MS was limited and TEs evaluated in the following for instrument comparison were considered only within the range of *m*/*z* 23 (^23^Na^+^) to 238 (^238^U^+^). For the ICP-TOF-MS, this mass range was acquired using a dwell time of 0.25 s, enabled by its much faster acquisition speed. While lower dwell times would be possible (and necessary) for single particle analysis, this study focused on total TE concentrations; therefore, a longer dwell time was prioritised to reduce the volume of acquired data significantly. After a take-up time of 1.5 min to reach signal stabilization, each sample was measured for only 1 min, still resulting in a much higher total of 242 measurements per mass peak compared to the ICP-SF-MS. The same 35 TEs as for the ICP-SF-MS were analysed, also measuring three replicates for each sample.

To ensure comparable conditions, the same inlet system (Apex Q, Elemental Scientific, Omaha NE, USA) equipped with a 100 μL microflow self-aspirating PFA nebulizer (Elemental Scientific, Omaha NE, USA) was used for both instruments. Droplets produced in the nebulizer were heated to 100 °C in a cyclonic spray chamber to evaporate the water, which was removed subsequently in a Peltier cooled condenser (2 °C). The wastewater was discharged through a separated waste capillary, while the resulting dry aerosol was injected into the plasma. Producing such a dry aerosol increases the sensitivity by a factor of 3–10 (ref. 31–33) and therefore lowers the limits of detection. For the analysis at both instruments, the nebulizer liquid flow was 400 μL min^−1^. Samples were introduced into the inlet system with an autosampler (CETAX ASX-260, Teledyne Cetac, Omaha NE, USA for the ICP-SF-MS; microDX, Elemental Scientific, Omaha NE, USA for the ICP-TOF-MS).

### Data processing, calibration, blank correction

The datasets from both, ICP-SF-MS and ICP-TOF-MS were processed identically. Raw intensity data were evaluated with a dedicated MATLAB (Mathworks Inc.) code. To remove outliers in the 14 (ICP-SF-MS) or 242 (ICP-TOF-MS) measurements per mass peak acquired for each sample, a Grubbs-test (4*σ* limit) was performed, before the average and the standard deviation for every isotope were calculated.^27^ The intensity of the internal ^103^Rh standard was used for normalization, to account for potential fluctuations of plasma conditions or nebulizing efficiency causing variations in the signal intensities.

For external calibration, the procedure from previous studies^27,34^ based on a dilution series (*n* = 7) of a multi-element standard (Spex CertriPrep, Inc., Metuchen, NJ, USA) was used. The concentrations of the TEs in the calibration standards were adjusted to cover their varying levels in glacier ice by diluting the stock solution from 1 : 1 to 1 : 2′000. The calibration function was obtained by linear regression and the corresponding correlation coefficients were generally higher than 0.9993.

Obtained concentration values were further processed, applying a blank correction, for both, analytical and procedural blanks. Analytical blanks (0.2 M HNO_3_ in UPW) were prepared daily to determine the background contamination of the current laboratory process. Procedural blanks (frozen UPW) were treated like ice samples (see samples and sample preparation) to determine potential contamination introduced during sample preparation. For the blank correction daily averages of at least 9 analytical blanks were used. Mean procedural blanks were determined from a total of 40 frozen UPW blanks for all elements except Na, Al, Ca, Cu, and Ba. For these TEs the procedural blanks varied from cutting session to cutting session and for correction individual blanks were used.

Blank-corrected concentration values below the method limit of detection were replaced by half of the LoD value. For each instrument, the LoD was defined as three times the standard deviation of all analytical blank measurements, following the removal of statistical outliers from the blank ensemble using the interquartile range (IQR) method. The analytical precision depends on the concentration level and is here reported as an average value over the concentration range defined by the mean concentrations measured in the two ice cores (Colle Gnifetti and Cerro Negro). The lower bound of this range is set to be at least three times the determined LoD. It was assessed by treating the daily measured dilutions of the multi-element standard within the defined concentration range like a sample during raw data post-processing. For the same concentration range, the instrumental precision was determined from the pooled standard deviation of triplet measurements performed on the ice core samples (same vial, measured sequentially). For determination of the analytical accuracy, a sample of a certified reference material (CMR; rainwater, TMRAIN-04, National Research Council of Canada, Burlington, Canada) was analysed at least once at the end of each measurement sequence, in a 20-fold dilution to result in a concentration range comparable to the one in ice core samples.

To investigate the agreement between the ice-core TE concentrations obtained with the ICP-SF-MS and the ICP-TOF-MS, *t*-tests were used to compare the ice core concentration averages, and Pearson correlation analyses was conducted to compare the temporal variability of the records. For not normally distributed data, a *t*-test can be performed, when the number of samples is larger than 30.^35^ This was done on both ice core sections, although for Colle Gnifetti the number of samples is 26. For calculating the averages, a TOF-MS/SF-MS concentration pair was discarded if one or both values were ≤LoD, since the LoDs of the two instruments are different for all TEs. This concerns primarily the Cerro Negro ice core samples since TE concentrations are much lower than in the Colle Gnifetti core. For the Cerro Negro samples, a 3-point moving average was applied before correlation analyses to account for the less precise depth alignment. In addition, for selected TEs, Bland–Altman plots^36^ were produced to analyse in detail the agreement between the data sets obtained with the two instruments.

## Results and discussion

### Limits of detection, procedural blanks, and precision

Comparable LoDs were obtained with ICP-SF-MS and ICP-TOF-MS for most of the TEs (Table 2). Aside from differences in some TEs with particularly low LoDs in both instruments (*e.g.*, lanthanides), notable exceptions are Sc, V, Fe, and Ba, which may reflect differences in laboratory background conditions and/or different batches of auto-sampler vials. For Sc, the higher LoD of the TOF-MS was related to its lower mass resolution (insufficient separation from ^29^Si^16^O^+^ and ^90^Zr^2+^ interferences; see later discussion). The LoDs of the ICP-SF-MS are comparable to previous studies conducted with the same instrument.^9^ The LoDs of the ICP-TOF-MS are comparable to those reported by Willie *et al.* (2001),^37^ but are generally higher than in Erhardt *et al.* (2019):^19^ Na (56% higher), Mg (18%), Ca (92%), and Al (70%). Although the same instrument and data processing methods as in Erhardt *et al.* (2019) were used, differences in the set-up and in the determination of the LoDs can explain the observed discrepancy. Specifically, they applied a set-up for continuous flow analysis (CFA) and defined the instrumental LoD from the short-term variability (2 second measurement intervals) during acquisition of analytical blanks. CFA involves minimal sample handling and no ambient exposure of the sample, thereby reducing the risk of background contamination. In contrast, manual decontamination procedures are required for discrete samples as analysed in this study. Therefore, a more conservative definition of the LoD was selected, which also reflects day-to-day variability of analytical blanks, and accounts for any variability introduced from analyte preparation to calibration (method LoD; see Material and methods).

Limits of detection (LoD), procedural blanks and analytical precision obtained with ICP-SF-MS (SF) and ICP-TOF-MS (TOF). For the average concentrations of the two ice cores – Cerro Negro (CN) and Colle Gnifetti (CG) - provided with their standard errors, only values exceeding the LoD of both instruments were considered. Also presented are the correlation coefficients from the linear regressions between the concentration records derived from the SF and TOF instruments. All numbers are rounded to significant digits. See Tables S1 and S2 for instrumental precision and analytical accuracy, respectively

**Table:** 

Element	LoD [ng L^−1^]		Procedural blanks [ng L^−1^]	Precision^b^ [%]		Cerro Negro, CN (*n* = 112)			Colle Gnifetti, CG (*n* = 26)		
	SF	TOF	SF and TOF	SF	TOF	Avg (SF) [ng L^−1^]	Avg (TOF) [ng L^−1^]	*r*	Avg (SF) [ng L^−1^]	Avg (TOF) [ng L^−1^]	*r*
^23^Na	207	209	a	6	13	3′300 ± 500	4′100 ± 400	0.13	110′000 ± 26′000	120′000 ± 25′000	0.96
^24^Mg	57	57	120 ± 20	5	8	1′300 ± 150	2′200 ± 220	0.87	100′000 ± 27′000	120′000 ± 34′000	0.96
^27^Al	179	244	a	3	2	14′800 ± 1′900	16′200 ± 1′700	0.88	190′000 ± 51′000	200′000 ± 50′000	0.99
^44^Ca	280	470	a	8	6	5′100 ± 750	7′600 ± 1100	−0.16	870′000 ± 270′000	900′000 ± 270′000	0.89
^45^Sc	0.2	11	<LoD	5	15	9 ± 2	31 ± 3	0.75	40 ± 9	140 ± 30	0.95
^51^V	7.8	30	<LoD	8	8	65 ± 7	72 ± 8	0.88	730 ± 150	730 ± 130	0.98
^52^Cr	1.5	3.0	4.8 ± 1.8	7	8	24 ± 3	28 ± 6	0.39	380 ± 90	390 ± 90	0.97
^55^Mn	2.3	5.4	6.3 ± 3.3	4	8	140 ± 13	170 ± 18	0.88	4′900 ± 1′200	5′000 ± 1′200	0.99
^56^Fe	33	113	<LoD	6	8	17′000 ± 2′300	18′000 ± 2′000	0.93	150′000 ± 38′000	140′000 ± 32′000	0.97
^59^Co	0.25	0.28	0.25 ± 0.05	5	7	4.1 ± 0.4	5.2 ± 0.5	0.74	110 ± 20	100 ± 20	0.96
^60^Ni	2.7	1.7	6.3 ± 2.5	4	4	18 ± 2	22 ± 4	0.82	300 ± 50	300 ± 50	0.96
^63^Cu	4.2	2.0	a	9	11	120 ± 12	120 ± 12	0.85	330 ± 60	330 ± 60	0.85
^66^Zn	220	330	<LoD	15	18	470 ± 80	750 ± 170	0.13	1′400 ± 160	1′500 ± 180	0.88
^85^Rb	0.73	0.62	2.7 ± 0.5	5	5	30 ± 3	40 ± 4	0.91	390 ± 110	380 ± 90	0.97
^88^Sr	1.13	1.1	5.0 ± 1.8	8	5	26 ± 3	52 ± 4	0.83	4′500 ± 1′500	4′600 ± 1′600	1.00
^90^Zr	0.27	0.68	9.4 ± 3.0	6	17	5.9 ± 0.7	6.7 ± 1.0	0.80	140 ± 30	150 ± 40	1.00
^95^Mo	0.51	0.52	2.8 ± 0.3	8	9	3.3 ± 0.3	3.7 ± 0.5	0.49	20 ± 3	21 ± 3	0.96
^109^Ag	0.11	0.21	0.3 ± 0.1	21	23	2.1 ± 0.2	1.2 ± 0.1	0.62	2.9 ± 0.5	2.9 ± 0.4	0.98
^111^Cd	0.12	0.21	0.4 ± 0.3	8	7	0.5 ± 0.1	3.0 ± 1.1	−0.01	14 ± 2	15 ± 2	0.94
^121^Sb	0.70	0.54	2.9 ± 4.2	5	8	4.7 ± 0.6	5.2 ± 0.6	0.81	30 ± 5	30 ± 4	0.94
^133^Cs	0.12	0.13	0.3 ± 0.1	5	8	14 ± 2	15 ± 2	0.90	25 ± 6	24 ± 5	0.98
^138^Ba	2.4	10.5	a	6	5	82 ± 9	127 ± 10	0.75	2′900 ± 760	3′200 ± 820	0.99
^139^La	0.04	0.18	<LoD	5	10	3.8 ± 0.4	4.1 ± 0.4	0.84	170 ± 50	180 ± 50	0.99
^140^Ce	0.05	0.29	0.3 ± 0.2	4	9	14 ± 2	15 ± 2	0.87	420 ± 120	430 ± 110	0.99
^141^Pr	0.01	0.06	<LoD	3	10	2.3 ± 0.3	2.5 ± 0.3	0.87	50 ± 10	50 ± 10	1.00
^146^Nd	0.02	0.07	0.08 ± 0.03	5	13	13 ± 2	13 ± 1	0.89	200 ± 50	200 ± 60	0.99
^147^Sm	0.02	0.01	0.02 ± 0.004	4	14	3.8 ± 0.4	3.6 ± 0.4	0.87	45 ± 12	45 ± 12	0.99
^153^Eu	0.03	0.002	0.03 ± 0.01	9	16	1.1 ± 0.1	1.0 ± 0.1	0.75	11 ± 3	11 ± 3	1.00
^172^Yb	0.03	0.01	<LoD	8	10	0.8 ± 0.1	0.9 ± 0.1	0.84	11 ± 3	12 ± 3	0.99
^182^W	0.30	0.12	0.3 ± 0.2	9	17	1.4 ± 0.2	0.6 ± 0.1	0.08	6 ± 1	6 ± 1	0.98
^205^Tl	0.15	0.18	0.8 ± 1.2	5	24	0.7 ± 0.1	0.7 ± 0.1	0.80	5.1 ± 0.8	4.6 ± 0.7	0.98
^208^Pb	0.61	0.85	2.7 ± 1.3	12	17	24 ± 3	35 ± 3	0.82	690 ± 100	720 ± 110	0.99
^209^Bi	0.03	0.16	0.2 ± 0.2	11	25	1.3 ± 0.2	1.1 ± 0.1	0.86	3.8 ± 0.7	3.5 ± 0.6	0.94
^232^Th	0.04	0.05	0.04 ± 0.04	14	17	1.0 ± 0.1	0.8 ± 0.1	0.87	26 ± 7	24 ± 6	1.00
^238^U	0.10	0.04	<LoD	8	14	0.6 ± 0.1	0.5 ± 0.1	0.79	14 ± 3	14 ± 3	0.98

a For TEs susceptible to contamination, the procedural blanks varied from cutting-to-cutting session. For correction, individual blanks as listed below were thus used.

b Precision depends on concentration and is here shown for the range between CN and CG averages, with the lower limit constrained to be at least 3× the LoD (higher of the two).

**Table:** 

Element^a^	SF-CN	SF-CG	TOF-CN	TOF-CG
^23^Na	370 ± 140	410 ± 150	2100 ± 1700	<LoD
^27^Al	220 ± 40	430 ± 170	560 ± 150	<LoD
^44^Ca	670 ± 130	2500 ± 1400	5100 ± 2700	1900 ± 1000
^63^Cu	7 ± 2	15 ± 7	14 ± 9	33 ± 20
^138^Ba	4 ± 1	4 ± 1	52 ± 7	55 ± 6

Procedural blanks are in the range of the LoDs or slightly higher for most TEs (Table 2). Exceptions are Na, Al, Ca, Cu, and Ba, which are all susceptible to contamination by sample handling, and thus can vary strongly between the different sampling days. The analytical precision (see Material and methods) achieved with the ICP-SF-MS is in the few percent range for most TEs, and on average almost a factor of two higher compared to the ICP-TOF-MS precision (7 ± 4% SF *vs.* 11 ± 6% TOF; Table 2). The ICP-TOF-MS precision is also lower by around a factor of two than values reported in the literature,^37,38^ likely since the latter were determined for higher concentration levels. As a general trend it is observed for both instruments, that the lower the concentration range, the lower the achieved analytical precision. This is more strongly pronounced for the analysis by ICP-TOF-MS (*e.g.*, Ag, W, Tl, Bi). Also, for TEs prone to contamination and present at relatively low concentrations compared to the procedural blank, analytical precision was lower for both instruments (*e.g.*, Zn, Pb). For Sc, the analytical precision was found to be about three times lower for the ICP-TOF-MS (15% compared to 5% for the ICP-SF-MS; see later discussion). The instrumental precision – assessed in this study using sequential triplet measurements (see Material and methods) which excludes effects from variability in daily calibration or from differences in ambient background conditions – was high for both instruments: 3 ± 1% for the ICP-TOF-MS and 6 ± 2% for the ICP-SF-MS on average, respectively (Table S1). A higher instrumental precision of the ICP-TOF-MS was observed for all TEs, with the sole exception of Sc (see later discussion). The analytical accuracy investigated for the certified TEs in the rainwater reference material (TMRAIN-04) was in the same range as the analytical precision for the respective instrument, generally again higher for the ICP-SF-MS compared to the ICP-TOF-MS (Table S2). Comparable to the analytical precision, also the level of accuracy largely reflects the TEs signal-to-noise ratio, *i.e.*, its concentration in the reference water in relation to its susceptibility to contamination (typically reflected by a rather high method LoD). For example, illustrated if comparing Fe, Zn or Pb with U in Table S2.

In summary, excluding Sc and TEs with lower nominal masses than reported here, the results indicate that the instrumental performance of the ICP-TOF-MS is fully sufficient and equally suitable for TE analysis, compared to the traditionally used ICP-SF-MS, at least under the analytical methods and procedures applied in this study.

### Effects and strategy of sampling

#### Influence of acidification time

**Fig. 1:**
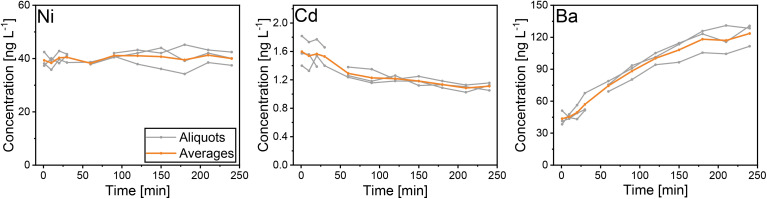
Acidification effect on ample concentration as a function of time. Concentrations of Ni, Cd and Ba are shown to represent TEs with constant, decreasing and increasing concentrations, respectively. Acidification times ranged from 1.5 min to 4 h. Individual aliquots are presented in grey and the average of all aliquots in orange.

Although the effect of acidification time was investigated on much shorter timescales than in previous studies,^11,12,14^ the results are in good agreement with those. The 35 analysed TEs can be categorised into four groups, based on their response to acidification over time (Fig. S1). A minority of four TEs (Co, Ni, Sb, Zn) displayed constant concentrations throughout the test phase. For seven TEs (Ag, Ca, Cs, Eu, Mn, Mo, Th), no clear pattern could be identified as their concentrations varied across different test days. The majority of TEs exhibited significant concentration changes, ranging from a decrease by −65% to an increase by +164% from the initial concentration after 1.5 min to the concentration after 4 h. Specifically, for eight TEs (Na, Cd, Tl, U, V, W, Yb, Zr) concentrations decreased with longer acidification times, while 16 TEs (Al, Ba, Bi, Ce, Cr, Cu, Fe, La, Mg, Nd, Pb, Pr, Rb, Sc, Sm, Sr) showed the opposite trend with increasing concentrations. Notably, for Ba and Mg the increase was larger than 100%. Examples for constant (Ni), decreasing (Cd), and increasing concentrations (Ba) with acidification times are given in Fig. 1. The increase in TE concentrations is caused by their leaching from particles. The decrease in TE concentrations observed upon acidification can be attributed to several potential mechanisms. One contribution factor is the formation of hardly soluble compounds, such as oxychlorides. These newly formed precipitates can subsequently adsorb onto sample tube walls or capillaries of the inlet system, effectively removing them from the analytical solution. Another potential mechanism involves the replacement of elements within the crystal lattice of minerals. For example, dissolved sodium ions can replace potassium ions within the muscovite crystal structure, subsequently causing that sodium is no longer available for analysis. Previous studies have also proposed mechanisms for concentration decreases upon acidification, including re-adsorption onto the mineral structure^14^ and the formation of new molecules through reactions between ions in the solution and the acid itself.^11^ In conclusion, even on short time scales, most of TE concentrations varied with acidification time. Thus, the shortest practicable acidification time of 1.5 min was applied in this study for comparing the two instruments. For future analyses of TE compositions of single particles, short acidification times are favourable since they leave the particles as unchanged as possible.

#### Effect of sample freezing

**Fig. 2:**
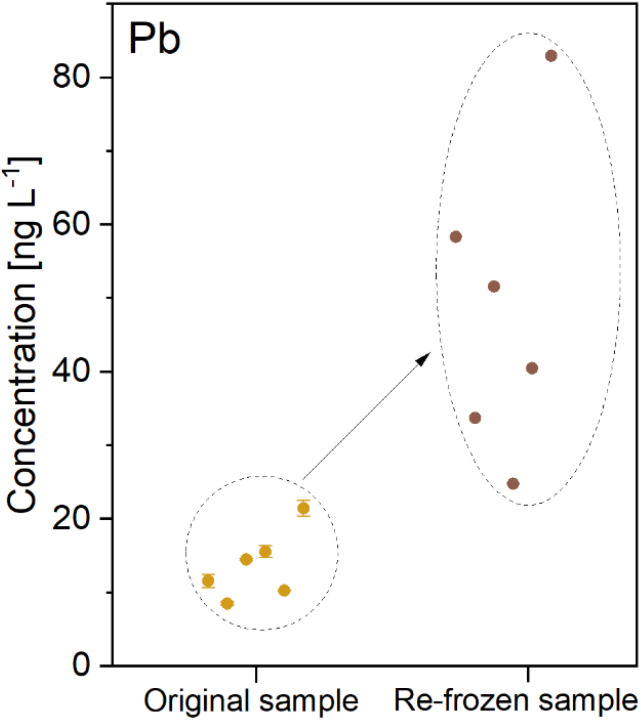
Refreezing effect on sample concentration for the example of Pb (error bars indicate the standard deviation of triplet measurements; invisible if smaller than the symbol size).

To analyse TEs in the same sample using both instruments located in different laboratories, the acidified samples needed to be refrozen and transported. Refreezing of samples resulted in significant discrepancies in concentrations for all TEs. As an example, the effect is shown for Pb in Fig. 2. These concentration changes followed the trends observed during the acidification test. During the freezing process, impurities are expelled from the ice, leading to steadily increasing acid concentration and ongoing leaching from particles in the remaining liquid part. As shown for the example of Pb, this consequently then results in an elevated concentration of the refrozen samples. Based on these findings, the finally selected approach to perform the comparability experiments with the ice core sections was to use parallel ice samples instead.

#### Inter-instrument comparability of ice sample concentrations

**Fig. 3:**
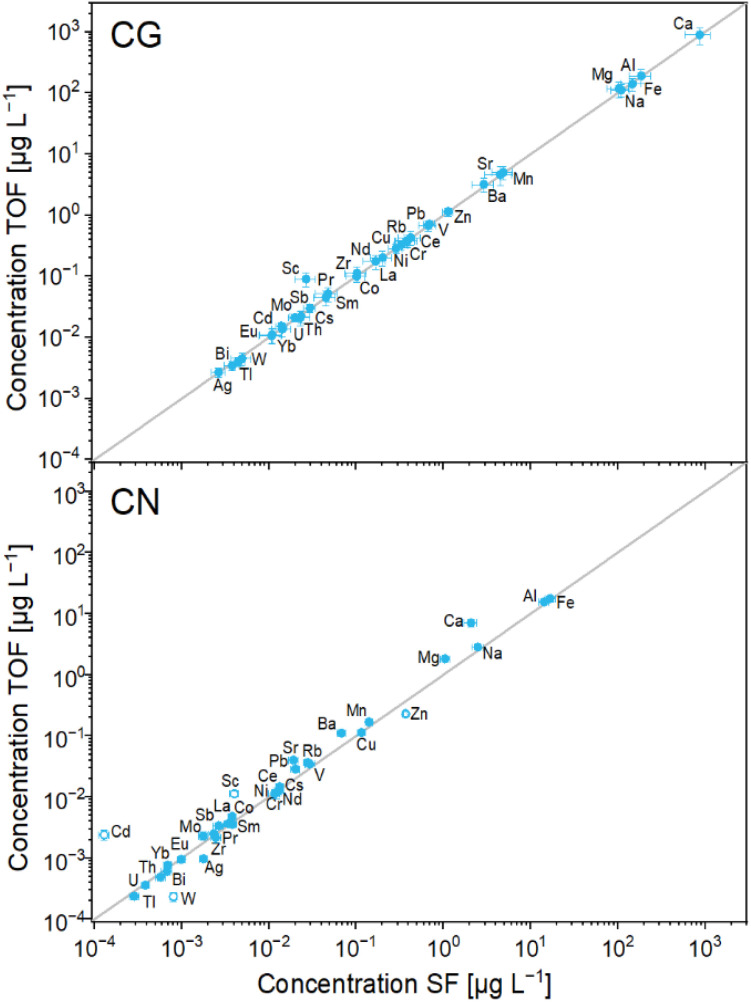
Scatterplot of TE average concentrations in the Colle Gnifetti (upper panel) and Cerro Negro ice core section (lower panel) obtained by ICP-TOF-MS and the ICP-SF-MS. Open symbols show TEs with less than 25% of sample pairs having concentrations above the LoD. Error bars denote the standard error. In grey, the 1 : 1 line is indicated.

**Fig. 4:**
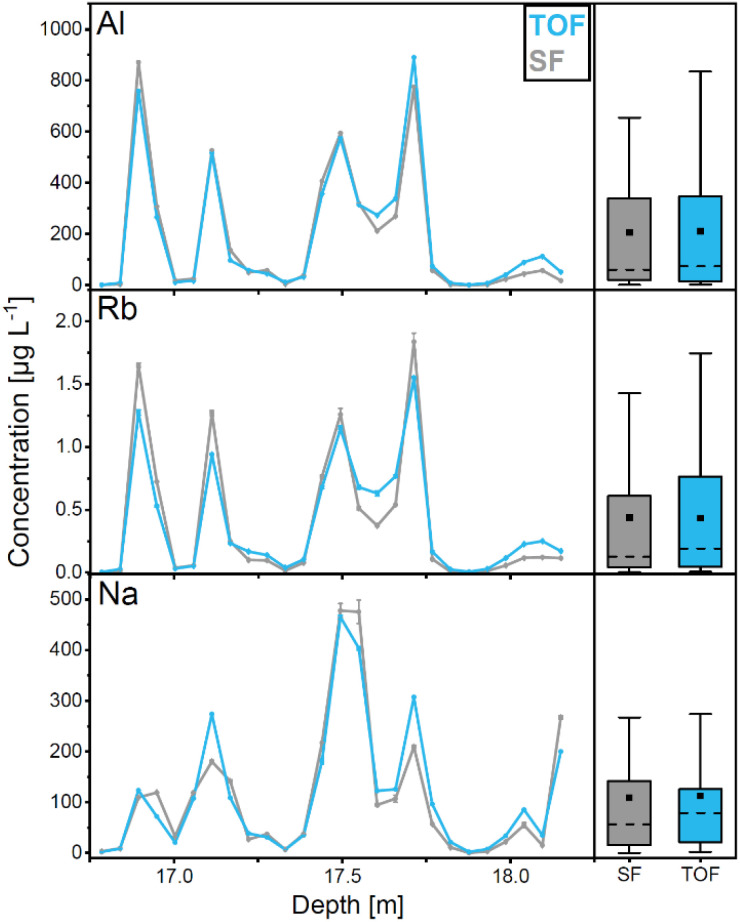
Concentration of Al (top), Rb (middle), and Na (bottom) in the Colle Gnifetti ice core section obtained with ICP-TOF-MS (blue) and ICP-SF-MS (grey). Error bars indicate the standard deviation of triplet measurements (small and thus invisible in most cases). The boxplots to the right, show the 5th, 27th, 75th, 95th percentiles, median (dashed) and average (square).

Average concentrations in the Colle Gnifetti samples obtained with ICP-TOF-MS and ICP-SF-MS are in exceptional correspondence for all TEs except Sc (Fig. 3 and Table 3), as indicated by the results of the *t*-test (*p* > 0.05). The agreement in the concentration variability along the ice core section is also excellent for all TEs, reflected by high correlation coefficients (*r* > 0.9) (Table 3). As examples, average concentrations and the concentration variability along the core section are shown for Al, Rb, and Na in Fig. 4.

Comparability of TE concentrations obtained with ICP-TOF-MS and ICP-SF-MS in samples from the Cerro Negro and Colle Gnifetti ice cores. For the Cerro Negro core, three-point moving averages were used for calculating the correlation coefficient (see Material and methods). For calculating the averages considered here, a TOF-MS/SF-MS concentration pair was discarded if at least one value was <LoD

**Table:** 

Cerro Negro (*n* = 112)		Colle Gnifetti (*n* = 26)
Comparability of temporal concentration variability with Pearson *r* analysis		
TE (#)	*r*	TE (#)
Fe, Rb, Cs (3)	>0.9	Na, Mg, Al, Ca, Sc, V, Cr, Mn, Fe, Co, Ni, Cu, Zn, Rb, Sr, Zr, Mo, Ag, Cd, Sb, Cs, Ba, La, Ce, Pr, Nd, Sm, Eu, Yb, W, Tl, Pb, Bi, Th, U (35)
		
Mg, Al, V, Mn, Ni, Cu, Sr, Zr, Sb, La, Ce, Pr, Nd, Sm, Yb, Tl, Pb, Bi, Th (19)	>0.8–0.9	
		
Sc, Co, Ba, Eu, U (5)	>0.7–0.8	
		
Ag (1)	>0.6–0.7	
		
Na, Ca, Cr, Mo, Zn, Cd, W (7)	<0.6	

a TEs with less than 25% of sample pairs having concentrations above the LoD.

**Table:** 

Comparability of average concentration with *t*-test		
TE (#)	*p*	TE (#)
Na, Al, V, Cr, Mn, Fe, Co, Ni, Cu, Rb, Zr, Mo, Sb, Cs, La, Ce, Pr, Nd, Sm, Eu, Yb, Tl, Bi, Th, U (25)	*p* > 0.05	Na, Mg, Al, Ca, V, Cr, Mn, Fe, Co, Ni, Cu, Zn, Rb, Sr, Zr, Mo, Ag, Cd, Sb, Cs, Ba, La, Ce, Pr, Nd, Sm, Eu, Yb, W, Tl, Pb, Bi, Th, U (34)
		
Mg, Ca, Sr, Ag, Ba, Pb (6)	*p* < 0.05	Sc (1)
		
aSc, Zn, Cd, W (4)	n.d.	

**Fig. 5:**
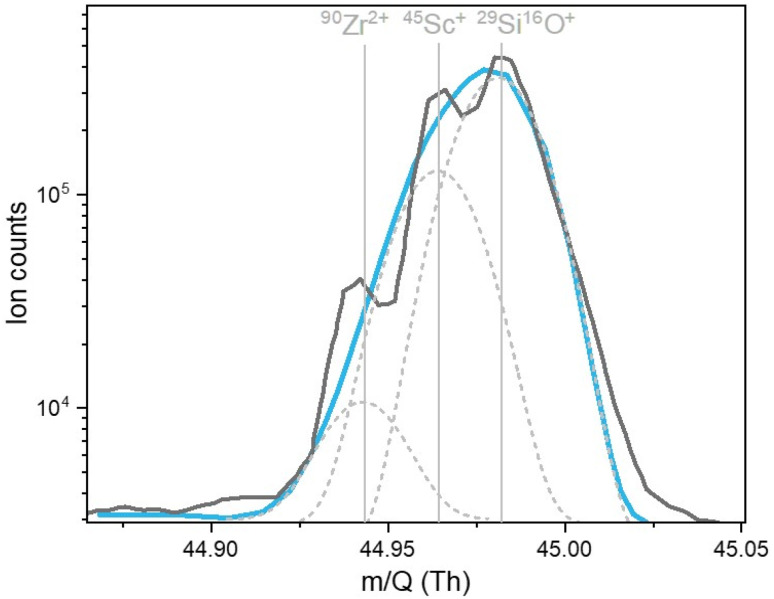
Scan of the analysis software TofDaqViewer (TOFWERK AG, Thun, Switzerland) showing the ^45^Sc^+^ peak not resolved from the peaks of the interferences ^90^Zr^2+^ and ^29^Si^16^O^+^. The dark grey curve is the recorded signal, representing the sum of the indicated three individual peaks (*i.e.*, masses; light grey dashed lines). The light blue curve is the smoothed signal identified as the ^45^Sc^+^ intensity peak by the software.

For Sc, the average concentration obtained with the ICP-TOF-MS is more than a factor of three higher than the one from the ICP-SF-MS, while the variability along the ice core section is similar (*r* = 0.95). As illustrated in Fig. 5, the nominal mass resolution of the ICP-TOF-MS of *m*/Δ*m* = 2′500–3′000 is not sufficient to fully resolve the ^45^Sc peak from the two interferences ^29^Si^16^O^+^ and ^90^Zr^2+^. For that a resolution of 2′902 and 12′628, respectively, would be required, or alternatively a suppression of them which has however shown to be particularly challenging.^39^ Across all samples and blanks, the intensity of the ^29^Si^16^O^+^ peak remained relatively stable, indicating that the quartz torch was the major source. However, because the concentrations of Zr and Si may vary from sample to sample – which is not the case for the calibration standards (and the CRM) – largely depending on the mineral dust content (*e.g.*, for Colle Gnifetti from silicate minerals in Saharan dust deposits), peak fitting procedures did not allow for satisfying precision, resulting in a general overestimation of Sc concentrations with the ICP-TOF-MS. For ^56^Fe the mass resolution of the ICP-TOF-MS (*m*/Δ*m* = 2′175) is nominally also not sufficient to separate the peak from the ^40^Ar^16^O interference, requiring *m*/Δ*m* = 2′503. The reaction of ^40^Ar^16^O with H_2_ significantly decreases the intensity of ^40^Ar^16^O and thus minimizes the interference.^40,41^ In this case the interference could sufficiently be suppressed through meticulous adjustment, involving fine-tuning the collision gas flow in the collision/reaction cell, which is filled with a 7% H_2_ in He gas mixture. In addition, concentrations of Fe were about four orders of magnitude higher than concentrations of Sc, resulting in a better peak fitting.

**Fig. 6:**
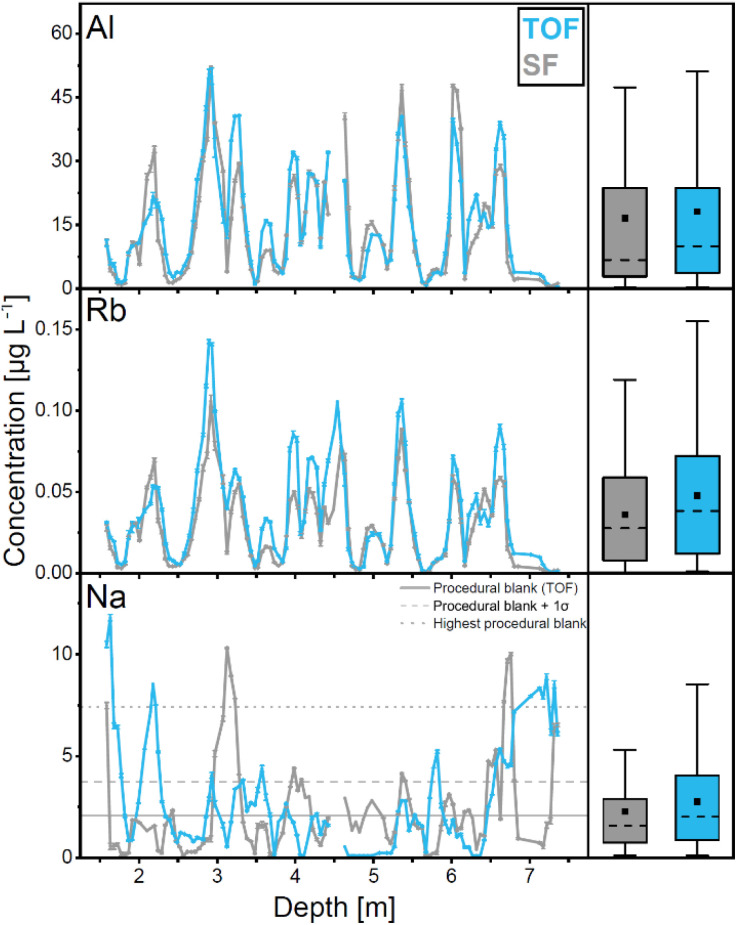
Concentration of Al (top), Rb (middle), and Na (bottom) in the Cerro Negro ice core section obtained with ICP-TOF-MS (blue) and ICP-SF-MS (grey). Error bars indicate the standard deviation of triplet measurements (small and thus invisible in most cases). The boxplots to the right, show the 5th, 27th, 75th, 95th percentiles, median (dashed) and average (square).

**Fig. 7:**
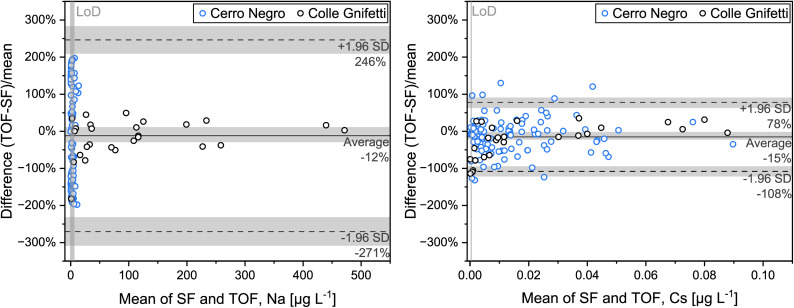
Bland–Altman plots showing the relative differences in Na (left) and Cs (right) concentrations between ICP-TOF-MS and ICP-SF-MS measurements for samples from Cerro Negro (blue circles) and Colle Gnifetti (black circles). Limits of detection (LoD) for Na and Cs are shown as vertical grey bars. The shown bias (solid line) and 95% limits of agreement (dashed lines), with grey shaded areas representing the respective 95% confidence intervals, were calculated using data from both ice cores. For Na, the limits of agreement are largely defined by values near the LoD. In contrast, Cs concentrations are mostly above the LoD, and although much lower on average, this still results in an overall higher correspondence.

For the Cerro Negro samples, TE concentrations obtained with ICP-TOF-MS and ICP-SF-MS are generally in good agreement, however, larger differences are observed for some TEs compared to the Colle Gnifetti samples. As will be discussed in more detail below, this can largely be attributed to the generally lower concentrations and potentially also to a less precise depth alignment of the parallel samples for this core. To reduce the impact of the latter in the evaluation of instrument comparability, a 3-point moving average was applied. The effect is illustrated for Al in Fig. S2. Average concentrations obtained with ICP-TOF-MS and ICP-SF-MS are in good agreement for 25 of 35 TEs (*p* > 0.05); see Fig. 3 and Table 3. For four of the TEs (Sc, Zn, Cd, W), less than 25% of the sample pairs showed concentrations above the LoD – Sc due to the high LoD caused by the interferences in the TOF-MS as discussed before – and these TEs will therefore not be discussed further. Of the 25 TEs with agreement in the average concentrations, 22 also show a similar concentration variability along the ice core section as indicated by correlation coefficients *r* > 0.7–0.9 (see *e.g.*, Al and Rb concentrations in Fig. 6). Rb concentrations are in the low ng L^−1^ range, but still well above the LoDs and the procedural blank. For the remaining three of these 25 TEs, the correlation coefficient *r* is lower than 0.6 (Na, Cr, Mo; Table 3). In contrast to Rb, Na concentrations in the samples from Cerro Negro are frequently close to the LoD. Na is further susceptible to contamination, reflected in variations of procedural blanks between the different cutting sessions, likewise observed for Al, Ca, Cu, and Ba. For blank correction of these TEs, individual blanks for each day were accordingly used (Table 2). Generally, our results suggest that for TEs with ice sample and procedural blank (and/or LoD) concentration levels being in the same order of magnitude, as it is the case *e.g.*, for Na, Cr and Mo, any observed discrepancies between ICP-TOF- and ICP-SF-MS should be attributed to the procedure rather than to differences in instrumental performance. In Fig. 6, this is illustrated on the example of the Na concentration record, which mostly varies within the range of the procedural blank concentrations. It becomes even more evident in the Bland–Altman plot (Fig. 7), showing that Na concentrations of most Cerro Negro samples are close to or even below the LoD (51% of sample pairs). As expected, these low concentration samples clearly show the largest relative concentration difference in measurements from the two instruments. For comparison, Fig. 7 includes a similar plot for Cs, for which concentrations in both cores are comparable and above the LoD for all samples. Some TEs like Na, Mg, Ca, or Ba, mostly present in water-soluble minerals, are prone to be relocated in glaciers affected by melt processes such as Cerro Negro.^9^ This might induce inhomogeneities between the parallel samples analysed by ICP-TOF-MS and ICP-SF-MS, respectively, additionally contributing to the reduced inter-instrument agreement for the samples from this site compared to those from Colle Gnifetti.

No agreement in the averages was observed for six of the TEs: Ca, Mg, Sr, Ag, Ba, and Pb (Table 3). However, for Mg, Sr, Ba, and Pb with concentrations above the LoD for ∼80% of sample pairs, relatively high correlation coefficients of *r* > 0.7 were still observed. The finding that ICP-TOF-MS averages are higher than ICP-SF-MS averages for most TEs (and for both cores), suggests that contamination control in the ICP-TOF-MS lab for discrete sampling was not optimal, particularly for the lower concentration range of the Cerro Negro ice core samples. This is not unexpected, as this lab is not fully equipped for manual decontamination under clean-room conditions, but was designed for CFA, which avoids any exposure of the melt fraction of the ice core samples from contact with the lab environment19. For Ag, the precision of the analysis is generally low for both instruments since concentrations are close to the LoDs.

## Conclusions

The performance of the new ICP-TOF-MS to determine concentrations of 35 TEs in ice core samples was tested by comparison with the established ICP-SF-MS technique. This aimed to investigate whether, for environmental samples – and particularly those at very low concentration levels – analysis by ICP-TOF-MS, with its benefit of additionally allowing single-particle analysis, can also yield the accuracy and precision for TE concentration measurements achieved by ICP-SF-MS, the technique traditionally used in the field of environmental and ice core research. Thereby the focus was on LoDs, precision, and comparability of TE concentrations in sections from two ice cores from the Colle Gnifetti and the Cerro Negro, characterized by impurity content in different concentration ranges. The two instruments were located at different institutions, about 100 km apart. Test for a suitable sampling strategy showed that concentrations of most TEs in ice samples varied with acidification time even on short timescales in the minutes range and refreezing of samples resulted in significant discrepancies in concentrations for all TEs. Thus, parallel samples from each core were prepared and analysed after an acidification time of 1.5 minutes. This is short enough to reduce wall effects but not strongly affecting the dissolution of particles of potential interest in future ice core studies for single particle analysis. Comparable LoDs were achieved with both instruments for most of the TEs. Exceptions are Sc, V, Fe, Ba, which may, for the latter three, reflect differences in laboratory background conditions and/or different batches of auto-sampler vials. For ^45^Sc the higher LoD of the TOF-MS is due to its lower mass resolution, resulting in insufficient separation from ^29^Si^16^O^+^ and ^90^Zr^2+^ interferences. The instrumental and analytical precision, as well as the achieved accuracy were found to be in the low percent range and comparable for the ICP-SF-MS and the ICP-TOF-MS.

For the ice core section with generally higher TE concentrations (Colle Gnifetti), average concentrations obtained with ICP-TOF-MS and ICP-SF-MS are in exceptional correspondence for all TEs (*p* > 0.05; except for Sc for reasons mentioned above). The agreement in the concentration variability along the ice core section is also excellent for all TEs as indicated by high correlation coefficients (*r* > 0.9).

For the ice core section with generally lower concentrations (Cerro Negro), average TE concentrations obtained with ICP-TOF-MS and ICP-SF-MS are generally also in good agreement, however, larger relative differences were observed for some TEs compared to the Colle Gnifetti samples. Larger differences were observed for TEs which are prone to contamination, especially if procedural blanks were challenging to control and varied over time (*e.g.*, Ba, Ca, Na). Because some TEs are also prone to be relocated in glaciers affected by melt like Cerro Negro, induced inhomogeneities between the parallel samples (in addition to a generally reduced precision in the depth alignment of samples from this core) may have further contributed to the observed differences between the two instruments. With concentrations in the samples from Cerro Negro close to the concentration levels of the procedural blanks or even the limit of detection for many of the TEs, larger relative discrepancies were also observed for *e.g.*, Ag, Cd, Cr, Mo, Pb, W, Zn. Low concentrations, associated with a reduction in analytical precision and a higher relative contribution from the procedural blank are thus rather responsible for the observed discrepancies between the results obtained with the two instruments than differences in their performance.

In summary, the performance of the new ICP-TOF-MS to determine concentrations of TEs in ice core samples was found to be excellent for the investigated trace elements within a nominal mass range from 23 to 238. Except for interferences on the ^45^Sc mass, which could not be resolved by the resolution of the instrument or sufficiently be suppressed in the collision/reaction cell of the TOF. For most applications in ice core research this is acceptable, especially when considering the great benefit to analyze single particles in the same sample.

## Author contributions

T. S. M. conducted the experimental work, performed data analysis, and wrote the manuscript. T. M. J. designed the experiments, contributed to the experimental work, supported or performed data processing and analysis, and assisted with data interpretation and manuscript writing. A. E. contributed to data analysis and interpretation. G. L. was involved in the experimental work at the ICP-TOF-MS and participated in the related data processing. M. S. conceived and designed the project, and contributed to interpretation, and manuscript writing.

## Conflicts of interest

There are no conflicts to declare.

## Supplementary Material

JA-040-D5JA00286A-s001

## Data Availability

Supplementary information is available. See DOI: https://doi.org/10.1039/d5ja00286a. For data presented in this article and its accompanying SI that are not provided in the tables, see: Mendeley Data at https://doi.org/10.17632/6th6y85gxt.1.
