# Supplementary material for: Performance of ICP-TOF-MS for ultra-trace element analyses in ice cores
Source: J Anal At Spectrom. 2025 Oct 6;40(12):3541–52. doi: 10.1039/d5ja00286a (PMC12551728; doi:10.1039/d5ja00286a)
Supplement: JA-040-D5JA00286A-s001 [file JA-040-D5JA00286A-s001.pdf]

Supplementary Information (SI) for Journal of Analytical Atomic Spectrometry.
This journal is © The Royal Society of Chemistry 2025

Supplementary Information

Performance of ICP-TOF-MS for ultra-trace element analyses in ice cores

T.S. Münster[abc], T.M. Jenk[ac] *, A. Eichler[ac], G. Lee[cd], M. Schwikowski[abc]

[a.] *PSI Center for Energy and Environmental Sciences, Villigen PSI, Switzerland, Department of Chemistry, Biochemistry and Pharmaceutical Sciences, University of Bern, Bern, Switzerland*
[b.] *Oeschger Centre for Climate Change Research, University of Bern, Bern, Switzerland*
[c.] *Climate and Environmental Physics, Physics Institute, University of Bern, Bern,* Switzerland
* *Corresponding author: Theo M. Jenk (theo.jenk@psi.ch*

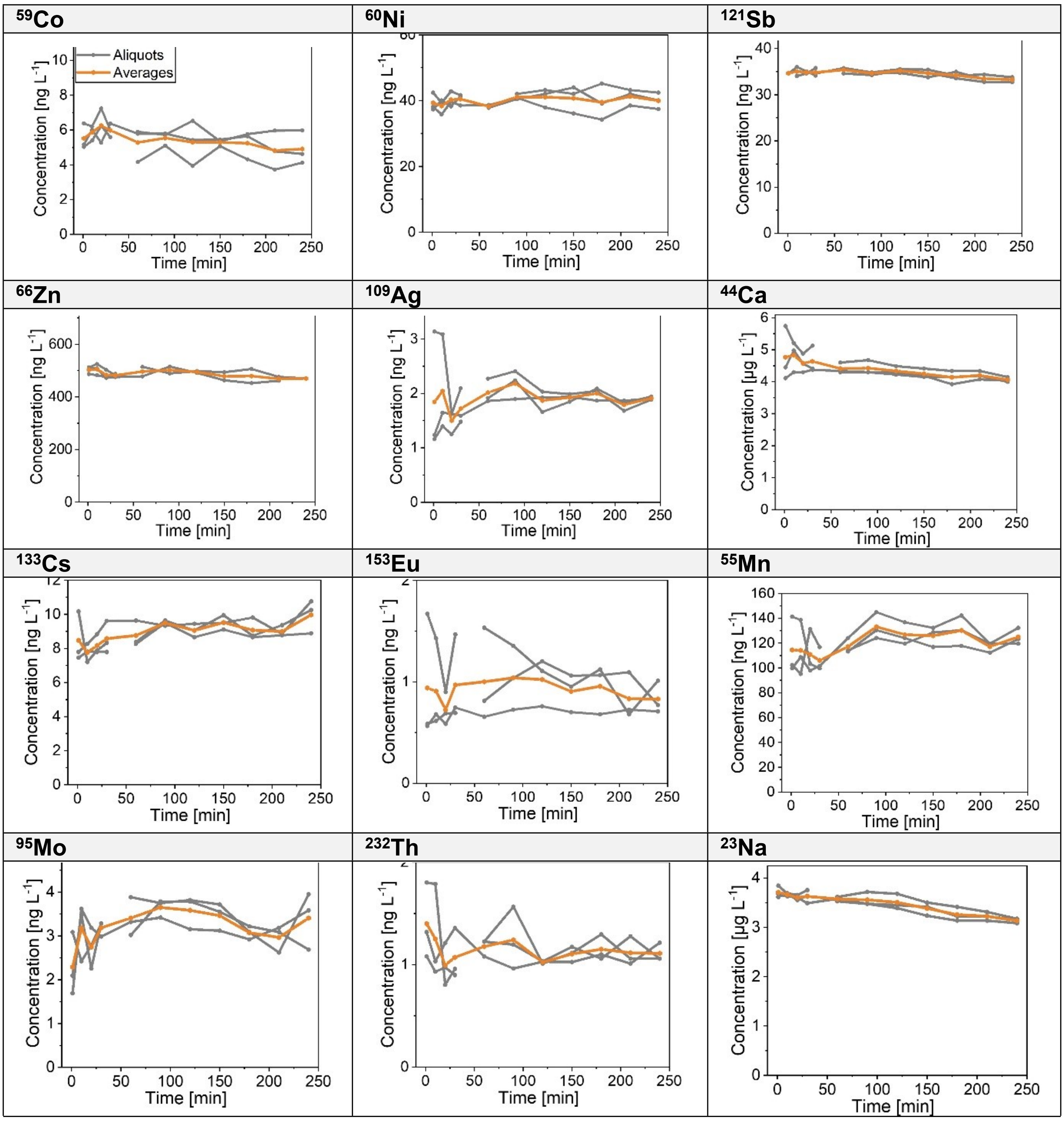

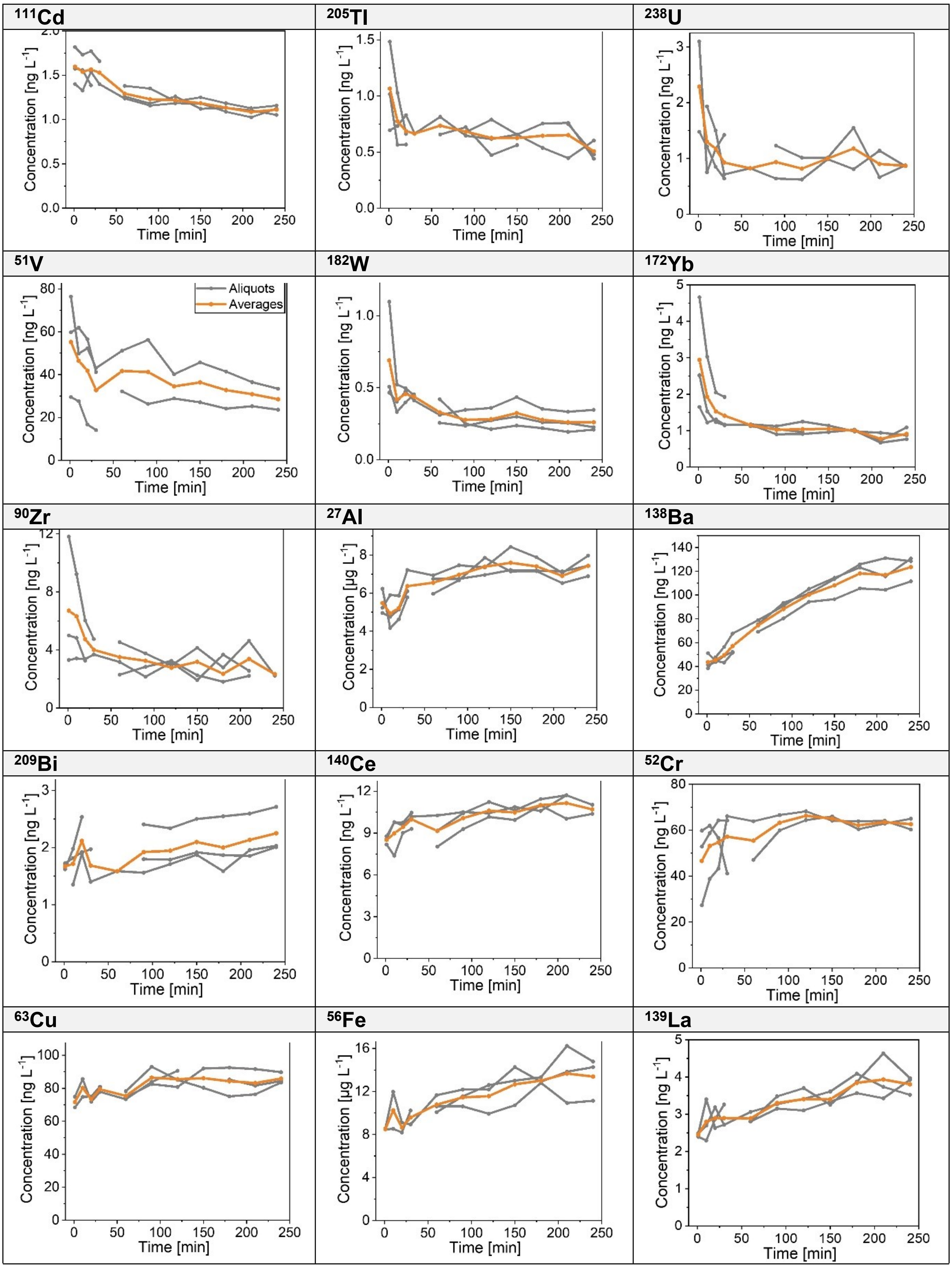

^{111}Cd
^{205}Tl
^{238}U
^{51}V
Aliquots
Averages
^{182}W
^{172}Yb
^{90}Zr
^{27}Al
^{138}Ba
^{209}Bi
^{140}Ce
^{52}Cr
^{63}Cu
^{56}Fe
^{139}La
Concentration [ng L^{-1}]
Concentration [µg L^{-1}]
Time [min]

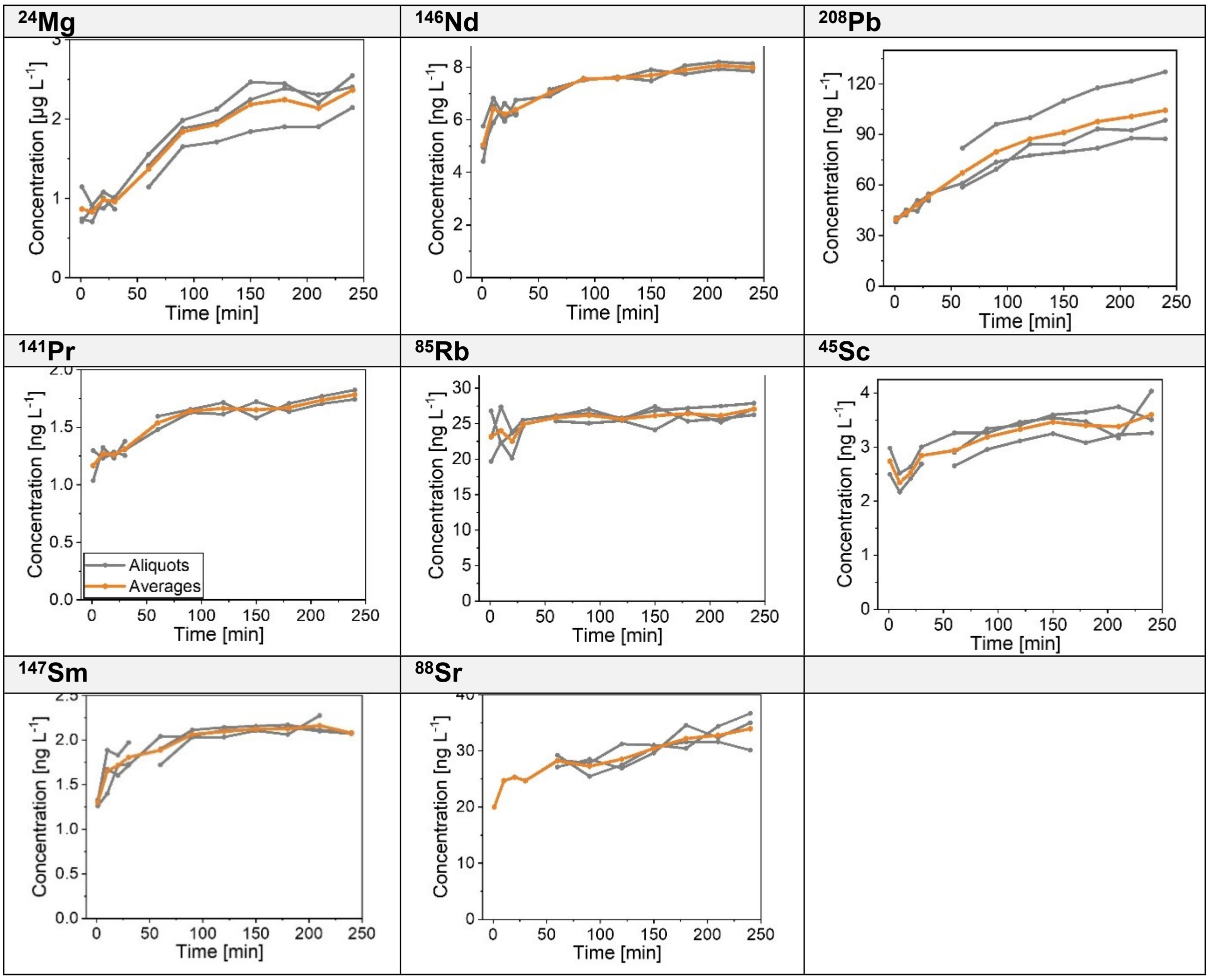

Figure S1: Concentrations of TEs with acidification times ranging from 1.5 min to 4 h. Shown are individual aliquots (grey) and the average of all aliquots (orange).

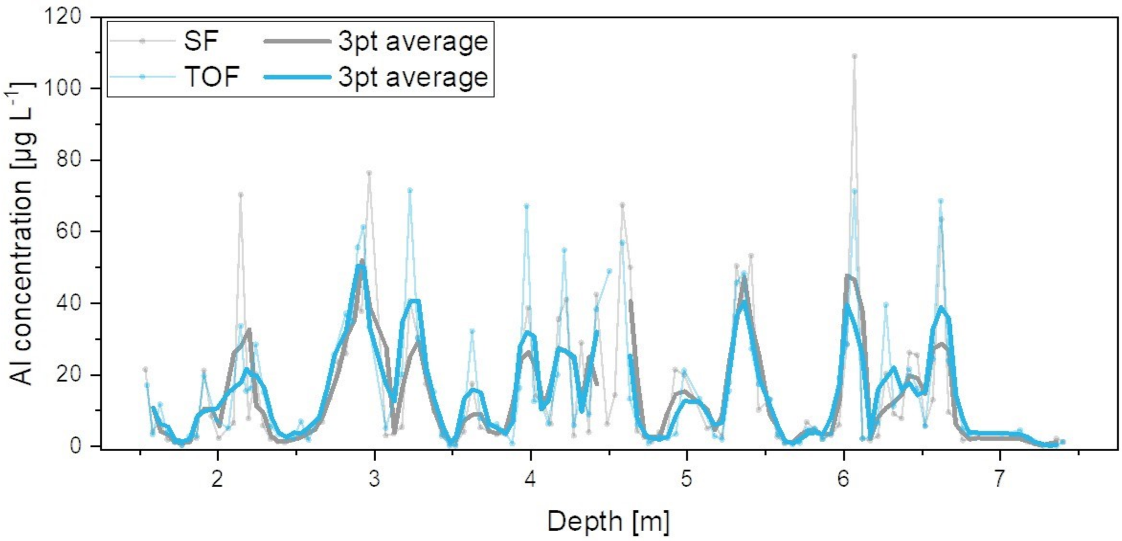

Figure S2: Comparison of raw data (light blue/light grey) and 3-point averages (bold lines, blue/grey) for Al concentrations in the Cerro Negro ice core measured by ICP-TOF-MS and ICP-SF-MS. The 3-point average was applied to compensate for inconsistent parallel sample cutting, resulting in improved alignment along the ice core depth.

Table S1: Instrumental precision, derived from the pooled standard deviation of all triplet measurements (from same vial, measured sequentially) performed for the Colle Gnifetti (CG) and Cerro Negro (CN) ice core samples. Provided values are representative for concentrations in the range between the CN and CG averages provided in the main manuscript (see Table 2), with the lower limit constrained to be at least three times the LoD (higher of the two; see Table 2). For analytical precision see Table 2 in the main manuscript, and Table S1 for analytical accuracy.

Element	Instrumental precision SF [%]	Instrumental precision TOF [%]
^{23}Na	5	2
^{24}Mg	5	3
^{27}Al	2	2
^{44}Ca	8	2
^{45}Sc	2	4
^{51}V	6	2
^{52}Cr	7	2
^{55}Mn	3	2
^{56}Fe	6	2
^{59}Co	5	2
^{60}Ni	4	2
^{63}Cu	4	1
^{66}Zn	7	2
^{85}Rb	4	2
^{88}Sr	8	3
^{90}Zr	6	4
^{95}Mo	8	5
^{109}Ag	9	3
^{111}Cd	3	3
^{121}Sb	5	2
^{133}Cs	5	2
^{138}Ba	4	3
^{139}La	4	2
^{140}Ce	3	2
^{141}Pr	3	2
^{146}Nd	3	2
^{147}Sm	4	2
^{153}Eu	7	2
^{172}Yb	8	2
^{182}W	9	3
^{205}Tl	6	3
^{208}Pb	11	4
^{209}Bi	11	6
^{232}Th	5	2
^{238}U	8	3

Table S2: Certified element concentrations in TMRAIN-04, element concentrations of the diluted reference water (dilution factor 21.1), and the according average concentrations as analysed by ICP-SF-MS and ICP-TOF-MS, respectively. Listed are all elements for which the concentration in TMRAIN-04 is certified. Shown uncertainties indicate the 95% confidence interval of the certified values for TMRAIN-04 (defined as: $\sigma * 1.96 / \sqrt{N}$; with population N varying for the different elements, ~60 on average), and the standard deviation of measured concentrations by ICP-SF-MS or ICP-TOF-MS, respectively. The provided accuracy denotes the percentual deviation of the measured from the reference value (not considering its confidence range, i.e. its uncertainty). For Al, the target concentration was below the LoD.

	TMRAIN-04		ICP-SF-MS (N=14)		ICP-TOF-MS (N=10)	
Element	Certified concentration [ng L^{-1}]	Reference concentration [ng L^{-1}]	Average concentration [ng L^{-1}]	Accuracy [%]	Average concentration [ng L^{-1}]	Accuracy [%]
Al	1940±80	92±4	<LoD	-	<LoD	-
V	672±13	31.9±0.6	32±2	0.4	34±4	6
Cr	861±22	41±1	44±4	8	44±7	7
Mn	6700±64	318±3	321±27	<1	324±40	2
Fe	24300±424	1153±20	1282±111	11	1304±240	13
Co	245±8	11.6±0.4	12±1	5	13±2	8
Ni	910±15	43.2±0.7	45±5	4	49±7	13
Cu	6950±80	330±4	352±23	7	343±60	4
Zn	8520±272	404±13	461±179	14	518±222	28
Sr	1810±33	86±2	86±5	<1	87±7	2
Mo	219±11	10.4±0.5	11±1	1	11±2	2
Cd	520±6	24.7±0.3	25±2	<1	26±9	6
Sb	345±10	16.4±0.5	17.9±0.5	9	18±2	12
Ba	868±14	41.2±0.7	43±2	5	56±22	35
Tl	371±11	17.6±0.5	18±1	<1	17±3	6
Pb	344±9	16.3±0.4	19±3	18	16±3	<1
U	293±4	13.9±0.2	14±1	<1	14±3	<1
